# Analysis of Draft Genome Sequences of Two New *Pantoea* Strains Associated with Wheat Leaf Necrotic Tissues Caused by Xanthomonas translucens Reveals Distinct Species

**DOI:** 10.1128/MRA.00638-20

**Published:** 2020-07-23

**Authors:** Alexandre Malette, Renlin Xu, Suzanne Gerdis, Sylvia I. Chi, Greg C. Daniels, Michael W. Harding, James T. Tambong

**Affiliations:** a Ottawa Research and Development Centre, Agriculture and Agri-Food Canada, Ottawa, Ontario, Canada; b Biotechnology Program, La Cité Collégiale, Ottawa, Ontario, Canada; c Crop Diversification Centre South, Alberta Agriculture and Forestry, Brooks, Alberta, Canada; University of Arizona

## Abstract

We report whole-genome sequences of two new *Pantoea* strains (DOAB1048 and DOAB1050) isolated from necrotic wheat leaves caused by Xanthomonas translucens. The draft genome sequences of DOAB1048 and DOAB1050 consist of 52 and 57 scaffolds and have sizes of 4,795,525 bp and 4,962,883 bp with 4,418 and 4,517 coding sequences, respectively.

## ANNOUNCEMENT

Wheat flag leaves exhibiting symptoms of bacterial streak caused by Xanthomonas translucens were collected, surface sterilized for 1 minute in 2% sodium hypochlorite, and rinsed three times with sterile water. Symptomatic leaves were aseptically excised, immersed in a 100-μl water droplet, and incubated for 5 minutes at room temperature. The suspension was streaked onto nutrient agar, and plates were incubated at 25°C for 48 h. Single colonies were obtained after repeated streaking (1). The bacterial isolates were cultured in Luria-Bertani broth overnight, and genomic DNA was isolated using the Wizard genomic DNA purification kit (Promega Corp.). Two isolates were characterized to the genus *Pantoea* by 16S rRNA sequencing (1, 2) and BLAST analysis (3). We report here the draft genome sequences of two *Pantoea* strains, DOAB1048 and DOAB1050. Libraries were constructed using a Nextera DNA Flex prep kit (Illumina) following the manufacturer’s instructions. The draft genome sequences were determined by paired-end sequencing using Illumina NextSeq technology at the Molecular Technologies Laboratory (Ottawa Research and Development Centre, Ottawa, Canada). A total of 1,250,924 and 1,459,666 paired-end reads, each 150 bp long, for strains DOAB1048 and DOAB1050, respectively, were generated. The quality of the reads was checked using FastQC version 0.11.3 (4), and reads were trimmed, if required. *De novo* assemblies were performed using ABySS version 1.5.2 (5) at k-mer values of 63 to 147. The best assemblies, based on *N*_50_ and *L*_50_ values and nearness to the expected genome size, were obtained at a k-mer value of 63, producing 52 and 57 scaffolds for strains DOAB1048 and DOAB1050, respectively. Scaffolds <300 bp long were discarded. The total sizes of the draft genomes are 4,795,525 bp (minimum, 1,382 bp; maximum, 303,105 bp; *N*_50_, 147,325 bp) and 4,962,883 bp (minimum, 607 bp; maximum, 561,133 bp; *N*_50_, 312,051 bp) for DOAB1048 and DOAB1050, respectively. The G+C contents of the draft genome sequences are 55.1% and 53.0%, and the overall estimated coverages were 38× and 44× for DOAB1048 and DOAB1050, respectively.

The draft genome sequences had no contamination and had a completeness of 100% based on PATRIC version 3.6.3 (Pathosystems Resource Integration Center) (6). The assembled sequences were annotated using the NCBI Prokaryotic Genome Automatic Annotation Pipeline (PGAAP), which identified 4,418 and 4,517 coding sequences, 45 and 57 tRNAs, 4 and 14 complete rRNAs, and 9 and 13 noncoding RNAs for DOAB1048 and DOAB1050, respectively. Prokka version 1.14.5 (7) annotated 1 transfer-messenger RNA (tmRNA) in the genome of each strain. Default parameters were used for all software unless otherwise specified. Strains DOAB1048 and DOAB1050 shared 2,925 PATRIC cross-genus protein families (PGfams) (6), with 847 and 875 unique PGfams, respectively. Genome-based *in silico* DNA-DNA hybridization (8) and average nucleotide identity (9) analyses taxonomically assigned strain DOAB1048 to Pantoea agglomerans, while DOAB1050 is a putative Pantoea allii strain.

### Data availability.

The whole-genome sequences are deposited in DDBJ/ENA/GenBank under the accession numbers JABLUT000000000 (DOAB1048) and JABLUS000000000 (DOAB1050). The versions described here are the first versions, JABLUT010000000 and JABLUS010000000. The raw reads are deposited in the NCBI Sequence Read Archive under the BioProject number PRJNA636100.
